# Structural and Optical Characterizations of Polymethyl Methacrylate Films with the Incorporation of Ultrafine SiO_2_/TiO_2_ Composites Utilized as Self-Cleaning Surfaces

**DOI:** 10.3390/polym15153162

**Published:** 2023-07-25

**Authors:** Maneerat Songpanit, Kanokthip Boonyarattanakalin, Saksorn Limwichean, Tossaporn Lertvanithphol, Mati Horprathum, Wisanu Pecharapa, Wanichaya Mekprasart

**Affiliations:** 1 College of Materials Innovation and Technology, King Mongkut’s Institute of Technology Ladkrabang, Bangkok 10520, Thailand; 64611004@kmitl.ac.th (M.S.); kanokthip.bo@kmitl.ac.th (K.B.); wisanu.pe@kmitl.ac.th (W.P.); 2 National Electronics and Computer Technology Center (NECTEC), National Science and Technology Development Agency (NSTDA), Pathum Thani 12120, Thailand; saksorn.limwichean@nectec.or.th (S.L.); tossaporn.ler@nectec.or.th (T.L.); mati.horprathum@nectec.or.th (M.H.)

**Keywords:** nanocomposite, polymethyl methacrylate, SiO_2_/TiO_2_ composite, sonochemical

## Abstract

The structural and optical characterizations of nanocomposite films of polymethyl methacrylate (PMMA) and SiO_2_/TiO_2_ composites prepared via the spin-coating technique were investigated using different SiO_2_:TiO_2_ ratios. The SiO_2_/TiO_2_ nanocomposites were synthesized using the sonochemical process with Si:Ti precursor ratios of 1:0.1, 1:0.5, 1:1, 1:2, 1:4, and 0:1. All characterizations of ultrafine SiO_2_/TiO_2_ particles were loaded at 1 wt.% in a PMMA matrix for the fabrication of transparent SiO_2_/TiO_2_/PMMA composite films. The phase structure and morphology of SiO_2_/TiO_2_/PMMA composite films were monitored using X-ray diffraction, optical microscopy, and field-emission scanning electron microscopy. A surface roughness analysis of SiO_2_/TiO_2_/PMMA nanocomposite films was conducted using atomic force microscopy. For optical characterization, transmission properties with different incident angles of SiO_2_/TiO_2_/PMMA composite films were analyzed with UV-vis spectrophotometry. The water contact angles of SiO_2_/TiO_2_/PMMA composite films were analyzed to identify hydrophilic properties on film surfaces. Photocatalytic reactions in SiO_2_TiO_2_ composite films under UV irradiation were evaluated using rhodamine B dye degradation. The optimal condition of SiO_2_/TiO_2_/PMMA nanocomposite films was obtained at a 1:1 SiO_2_:TiO_2_ ratio in self-cleaning applications, resulting from good particle dispersion and the presence of the TiO_2_ phase in the composite.

## 1. Introduction

Environmental challenges such as global warming, air pollution, and climate change are principal problems of global concern. Self-cleaning surfaces have shown great promise in recent years for being able to clean dust, organic contamination, and industrial pollutants on building surfaces, solar modules, and windshields [1]. The self-cleaning capacity of material surfaces contributes to environmental friendliness and cost reductions. Therefore, the improvement of new functional materials is of interest for research on self-cleaning applications using hydrophobic and hydrophilic mechanisms for contamination removal assisted by the action of water on the surfaces. For a hydrophilic surface, water droplets can spread over the entire surface to remove the contaminants, while a rough hierarchical structure with low surface energy has the capacity to induce the rolling down of water droplets and clean contaminants on its surface. Thus, the advantages of self-cleaning spreading water droplets on hydrophilic surfaces are useful, and these are appropriate for film coating on flat surfaces in the form of glazing decorations and sensors, including solar panels [2].

Among the potential materials with self-cleaning properties, silicon dioxide (SiO_2_) and titanium dioxide (TiO_2_) are remarkable metal oxide materials being considered in this specific field. SiO_2_, or silica material, is normally utilized in hydrophobic self-cleaning applications due to its exceptionally advantageous properties, including coverage of large surface areas, low-cost chemical inertness, high thermal resistance, and robust mechanical strength [3]. Moreover, SiO_2_ material can be modified via incorporation with other nanomaterials and into polymer matrices to produce nanocomposite films. Improvements in self-cleaning properties utilizing hierarchical micro/nanostructures’ wettability could be achieved with the incorporation of a silicon dioxide and polymer matrix, such as polydimethylsiloxane (PDMS), via the lower surface energy and good hydrophobicity on the composite film being modified by a high bond energy and wide bond angles in SiO_2_ chemical species and CH_3_ groups in PDMS chains [4]. TiO_2_, or titania material, is a promising metal oxide material with excellent hydrophilicity, photocatalytic activity, chemical stability, and environmental friendliness [5]. The prominent features of TiO_2_ material in terms of self-cleaning include the split of organic pollutants by photocatalytic mechanisms under ultraviolet activation and water spreading over the entire surface owing to the material’s hydrophilicity [6]. Therefore, combining nanocomposites with SiO_2_ and TiO_2_ materials can enhance self-cleaning reactions with the assistance of photocatalyst mechanisms. Decreased water-droplet contact angles and the need for hydrophilicity maintenance of TiO_2_ in a dark place or without UV irradiation could be obtained by increasing the hydroxyl content generated by incorporating SiO_2_ into a SiO_2_/TiO_2_ composite [7]. However, there are many methods for the synthesis of SiO_2_/TiO_2_ nanocomposites, such as sol–gel [8], hydrothermal [9], and sonochemical techniques [10], which result in different morphologies of synthesized SiO_2_/TiO_2_ nanocomposites. SiO_2_/TiO_2_ nanocomposites with different SiO_2_ contents, prepared via a sol–gel method accompanying an annealing process at 600 °C for 5 h, were reported by Manh et al. [11]. The results revealed that the inhibition in the anatase-to-rutile phase transition in the TiO_2_ matrix occurred due to the influence of the amorphous SiO_2_ surface layer suppressing the diffusion of anatase-phase particles in direct contact and limiting the ability of surface nucleation sites to progress to the rutile phase. Masanori and coworkers reported the synthesis of SiO_2_/TiO_2_ composite nanoparticles via a hydrothermal process operated at 200 °C [12]. Although the sol–gel-based method is simpler for synthesizing a SiO_2_/TiO_2_ composite structure, it requires post-thermal treatment, resulting in particle aggregation. On the other hand, the sonochemical process is one of cavitation, with the rapid growth and collapse of implosion bubbles in a liquid under high temperature and high pressure in the reaction environment, resulting in the formation of nanomaterials. The advantages of the sonochemical process include a short reaction time and facile control of size, with ultrafine particles at the nanoscale, as well as crystallinity and morphology [13]. Reactive species such as •OH and H_2_O_2_ can be created using the sol–gel method, accompanying sonochemistry based on the yield of homogeneous synthesis. The formation of nanocomposite film in the form of a polymeric matrix material with polymethyl methacrylate is well-known in acrylic resins and has such properties as a high strength and good dimensional stability, thermal stability, and outdoor wear [14]. A fabricated transparent nanocomposite film of PMMA as a matrix incorporated with nanoparticles acting as a self-cleaning layer has been successfully prepared using a dip-coating technique. However, using nanopowder products on self-cleaning surfaces is still a challenging task in the structural design, depending on the substrate surface, cost, and film thicknesses, when using different coating processes. Several methods for nanomaterials’ deposition on the substrate include dip coating, spray pyrolysis, sputtering, and spin coating [15]. Among them, spin coating is a facile and cost-effective method for depositing nanomaterial film on the substrate since the film thickness can be readily controlled by the spin speed [16,17].

In this study, we were interested in the synthesis of SiO_2_/TiO_2_ nanocomposite material via the sonochemical process. Meanwhile, the cooperation of SiO_2_/TiO_2_/PMMA nanocomposite films was fabricated using the spin-coating process. The effect of the SiO_2_/TiO_2_ nanocomposite distribution on morphologies, optical properties, hydrophilicity, and photocatalysis was studied. The investigation of the self-cleaning and photocatalytic properties of the prepared films under UV activation shows the difference in surface morphology and particle distribution depending on the TiO_2_ ratio in SiO_2_/TiO_2_/PMMA nanocomposite film.

## 2. Materials and Methods

**Figure 1:**
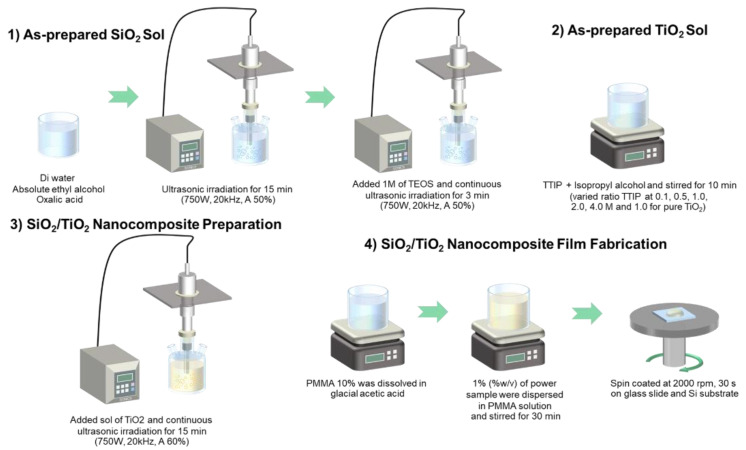
Schematic of SiO_2_/TiO_2_ nanocomposite synthesized via sonochemical process and SiO_2_/TiO_2_/PMMA nanocomposite films fabricated using spin-coating technique.

Polymethyl methacrylate (PMMA), tetraethyl orthosilicate (TEOS), and titanium isopropoxide (TTIP) were used as precursors in the sonochemical process for the synthesis of SiO_2_/TiO_2_ nanocomposite powder. The ratios of SiO_2_:TiO_2_ in SiO_2_/TiO_2_ nanocomposites were varied at 1:0.1, 1:0.5, 1:1, 1:2, 1:4, and 0:1 (pure TiO_2_). For SiO_2_/TiO_2_ nanocomposite synthesis, tetraethyl orthosilicate was slowly dropped in absolute ethyl alcohol, distilled water, and oxalic acid under sonochemical reaction for 15 min at 750 W, 20 kHz, and 50% amplitude to produce SiO_2_ sol. After that, TiO_2_ sol prepared by the solution of TTIP in isopropyl alcohol was mixed in SiO_2_ sol under an ultrasonic sonicator for 15 min at 750 W, 20 kHz, and 60% amplitude. Then, SiO_2_/TiO_2_ nanocomposite in the white precipitate was washed with DI water until pH 7 and dried overnight at 100 °C. For film fabrication, a certain amount of SiO_2_/TiO_2_ nanocomposite at 1 wt.% for all conditions was loaded in PMMA solution to produce the transparent nanocomposite suspension under continuous magnetic stirring. SiO_2_/TiO_2_/PMMA nanocomposite films were prepared using the spin-coating method on glass slides and silicon substrate and baked at 100 °C for 10 min to obtain transparent film. The schematic preparation of nanocomposite powder via sonochemical process and film fabrication using the spin-coating technique is exhibited in Figure 1. For the characterization part, the phase identification and surface morphologies of SiO_2_/TiO_2_/PMMA nanocomposite films were investigated with the X-ray-diffraction technique (XRD; Rigaku SmartLab (Tokyo, Japan)), field emission scanning electron microscope (FE-SEM; Hitachi S-8030, Tokyo, Japan), atomic force microscopy (AFM; Hitachi 5300E), and upright microscope (Leica DM6 M, Wetzlar, Germany). The optical transmittance was examined with a universal measurement spectrophotometer (Agilent; Cary 7000, Santa Clara, CA, USA) in the 200–2000 nm wavelength range. The self-cleaning assessment was carried out via contact angle measurement (ramé-hart instrument co.). The photocatalytic activity of all nanocomposite film samples was evaluated by testing their photocatalytic degradation with a droplet of rhodamine B (RhB) solution at 10 µM on the nanocomposite film surface. Then, the film surface was baked for 10 min to obtain the pink stain on transparent films and tested under UV irradiation for 120 min. After that, the color change of the RhB stain on the photocatalyst films at 0 and 2 h under UV activation was measured using a UV-vis spectrometer in transmittance mode to compare the dye degradation activity. A high %T value denotes that the transparent film has a good performance in the dye degradation process. Based on this measurement, the percentage of dye degradation was calculated following Equation (1)

(1) $$\%dye\;degradation=\frac{T_{2}-T_{0}}{T_{0}}\times 100$$

where *T*_0_ and *T*_2_ are %transmission at 0 and 2 h of UV light exposure.

## 3. Results and Discussion

### 3.1. Morphological and Structural Characteristics

**Figure 2:**
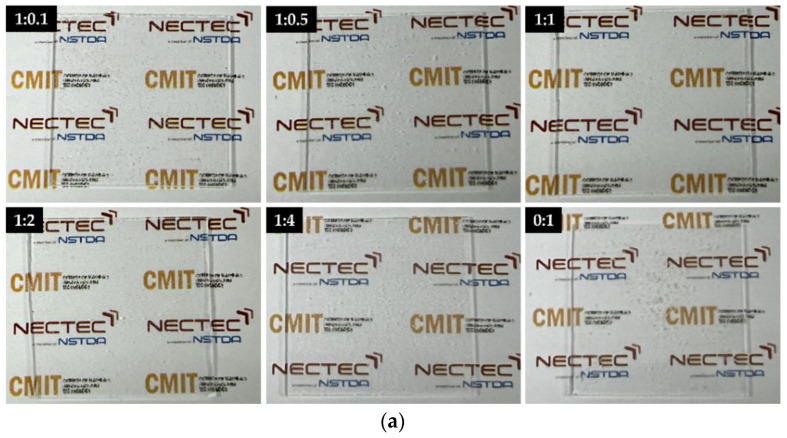

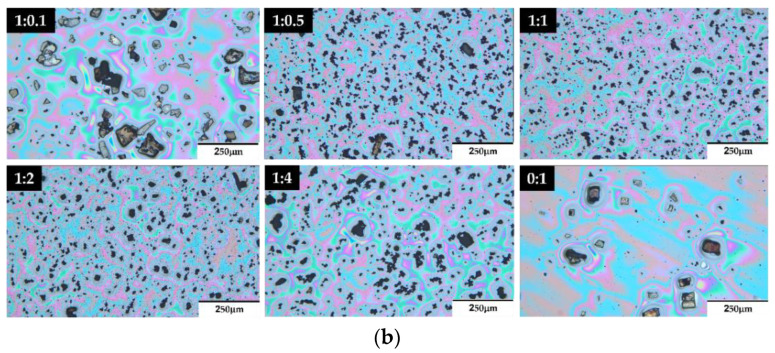
(**a**) Photographs and (**b**) optical images of transparent SiO_2_/TiO_2_/PMMA nanocomposite films with different SiO_2_:TiO_2_ ratios of 1:0.1, 1:0.5, 1:1, 1:2, 1:4, and 0:1.

All SiO_2_/TiO_2_/PMMA nanocomposite films showed SiO_2_/TiO_2_ nanocomposite powder on the PMMA matrix, as illustrated in Figure 2. As shown in Figure 2a, the particulate SiO_2_/TiO_2_ composites with PMMA matrixes were covered on the glass substrate with a highly transparent appearance. However, the aggregation of SiO_2_/TiO_2_ nanocomposites was observed in specific areas on the film surface due to the different TiO_2_ ratios in the composites. The optical micrographs were taken to monitor the morphology of SiO_2_/TiO_2_/PMMA nanocomposite films, as shown in Figure 2b. High-resolution images exhibited various particle sizes and various shapes of nanocomposite clusters on PMMA matrix film caused by the increase in TiO_2_ loading in the SiO_2_/TiO_2_ nanocomposite. For a low TiO_2_ content in SiO_2_/TiO_2_ nanocomposites with SiO_2_:TiO_2_ ratios of 1:0.1 and 1:0.5, more dense areas of particle aggregation with a square shape on the film surface were observed due to TiO_2_ gathering in SiO_2_ as a main material. In addition, SiO_2_/TiO_2_ nanoparticle distribution was improved by the increase in TiO_2_ loading in the SiO_2_:TiO_2_ ratio until 1:1. The appearance of this condition reveals the uniformity, small particle size, and good powder dispersion of SiO_2_/TiO_2_ nanocomposites covering the whole glass-slide substrate.

**Figure 3:**
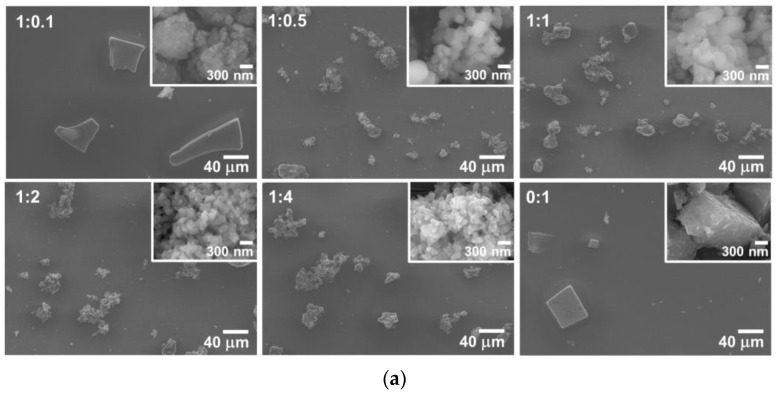

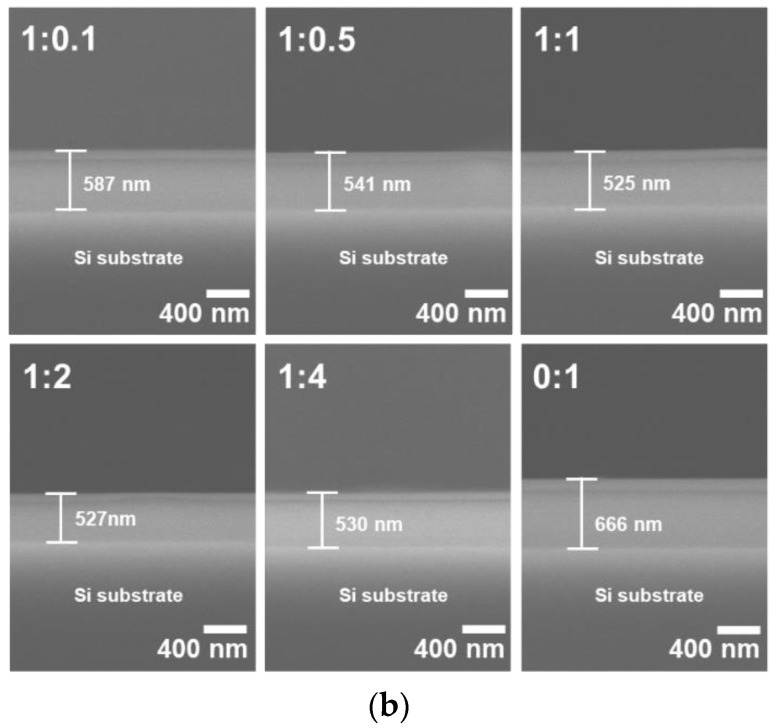
FE-SEM images of SiO_2_/TiO_2_/PMMA nanocomposite films with the different SiO_2_:TiO_2_ (**a**) surface morphologies and (**b**) cross-section images.

The surface morphologies and cross-section images of SiO_2_/TiO_2_/PMMA nanocomposite films were monitored using a field emission scanning electron microscope, as shown in Figure 3. This technique was chosen as the comparison, with optical micrographs to confirm the feature of the particle on the PMMA film surface. The SiO_2_/TiO_2_ composite powder was formed on the PMMA film surface with differences in size and shape due to the different forms of TiO_2_ loading in the composite, as shown in Figure 3a. For low TiO_2_ loading in the composite, particulate aggregation of SiO_2_ powder was obviously noticed. Meanwhile, the increase in particle distribution on PMMA film was obtained at the optimized SiO_2_:TiO_2_ ratio of 1:1. Therefore, the good particle distribution and decrease in the aggregation in the SiO_2_/TiO_2_ nanocomposite could be enhanced by the appropriate composite ratio of SiO_2_:TiO_2_, owing to the prevention of TiO_2_ crystallite growth with the influence of the SiO_2_ phase in nanocomposite synthesis under sonochemical processes [18]. However, the high content of TiO_2_ ratio in the nanocomposite could result in an increase in particle agglomeration, as seen in the SiO_2_:TiO_2_ composite ratios of 1:2 and 1:4. This result can be described by the facile TiO_2_ self-aggregation for high content in SiO_2_/TiO_2_ nanocomposites. Meanwhile, the average thickness of the SiO_2_/TiO_2_/PMMA nanocomposite film was analyzed using cross-section images, as depicted in Figure 3b. The values of nanocomposite film thickness were obtained at 587, 541, 525, 527, 530, and 666 nm for the films fabricated with SiO_2_:TiO_2_ ratios of 1:0.1, 1:0.5, 1:1, 1:2, 1:4, and 0:1, respectively. The difference in film thickness with various TiO_2_ loadings in the SiO_2_ composite could originate from the viscosity in the PMMA matrix with different particle sizes and particulate agglomerations.

**Figure 4:**
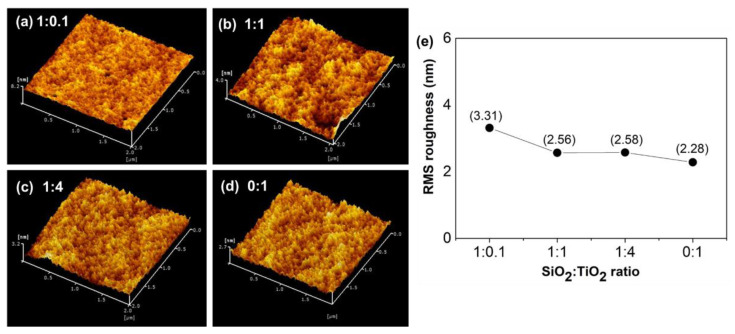
3D-AFM images of SiO_2_/TiO_2_/PMMA nanocomposite films with SiO_2_:TiO_2_ ratios of (**a**) 1:0.1, (**b**) 1:1, (**c**) 1:4, (**d**) 0:1, and (**e**) RMS roughness on the films with different SiO_2_:TiO_2_ ratios.

The three-dimensional AFM images of SiO_2_/TiO_2_/PMMA nanocomposites films with different SiO_2_:TiO_2_ nanocomposite ratios are shown in Figure 4. The morphological area of the film surface obtained with the AFM technique was studied on the smooth and transparent region without particle aggregation, with a length area of 2 µm × 2 µm. According to the FE-SEM results, surface morphologies on the film’s surface are shown as large-scale areas with the nanocomposite powder on PMMA film. Therefore, the smooth area on the film surface was further characterized to confirm the incorporation of particles and the polymer matrix. The surface roughness of the SiO_2_/TiO_2_/PMMA composite films revealed by AFM images possessed a similar surface structure regarding morphological homogeneity, as observed in Figure 4a–d. Moreover, the root mean square (RMS) surface roughness of all SiO_2_/TiO_2_/PMMA composite films is presented in Figure 4e. The greatest roughness with an RMS value of 3.31 nm was obtained as the film was filled with the composite with a SiO_2_:TiO_2_ ratio of 1:0.1 due to the large particle of the SiO_2_ matrix. The lowest RMS roughness value of 2.56 nm was monitored in the sample with a SiO_2_:TiO_2_ ratio of 1:1, in good accordance with the optical image and FE-SEM results.

**Figure 5:**
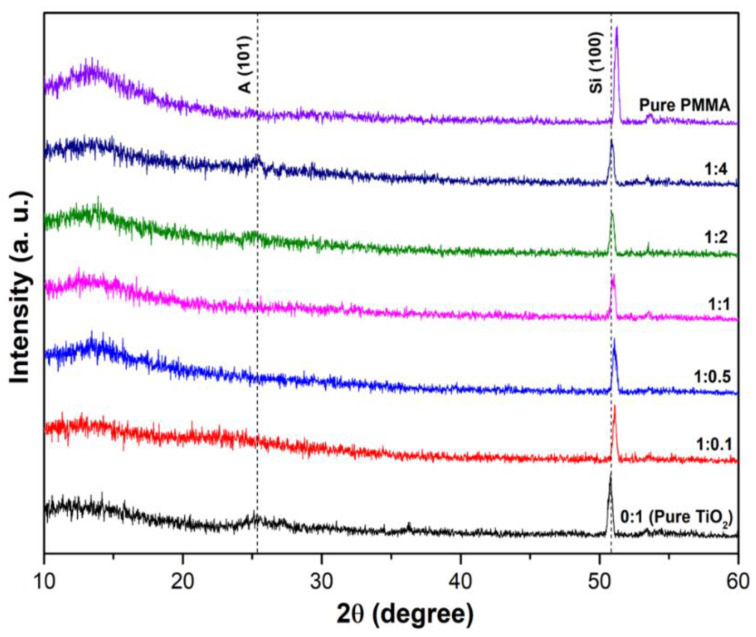
XRD patterns of SiO_2_/TiO_2_/PMMA nanocomposite films with different SiO_2_:TiO_2_ ratios.

The diffraction patterns of SiO_2_/TiO_2_/PMMA nanocomposites films on silicon substrates with different TiO_2_ ratios are shown in Figure 5. The diffraction patterns of pure TiO_2_ powder in the PMMA matrix (the sample with 0:1) positioned at 25.4°and 36° are attributed to the crystalline planes of the (101) and (103) anatase TiO_2_ phase (CSD No. 9008216). Meanwhile, the broad characteristic peak in the range from 15° to 25°, as noted in SiO_2_/TiO_2_ composite films with SiO_2_:TiO_2_ ratios of 1:0.1, 1:0.5, 1:1, 1:2, and 1:4, was associated with the SiO_2_ amorphous phase [19,20]. Moreover, the excessively strong intensity at 2θ of 50° is ascribed to the (100) crystalline planes of silicon wafer substrate [21]. The increase in TiO_2_ loading with the ratios of 1:2 and 1:4 led to the appearance of a noticeable peak at 2θ = 25.4° regarding ultrafine particles and the existence of TiO_2_ phase formation. Therefore, the XRD results of SiO_2_/TiO_2_/PMMA composite films imply that the formation of a strong TiO_2_ crystalline phase could possibly reduce he formation of the SiO_2_ phase in SiO_2_/TiO_2_ composites.

### 3.2. Optical Characteristics

**Figure 6:**
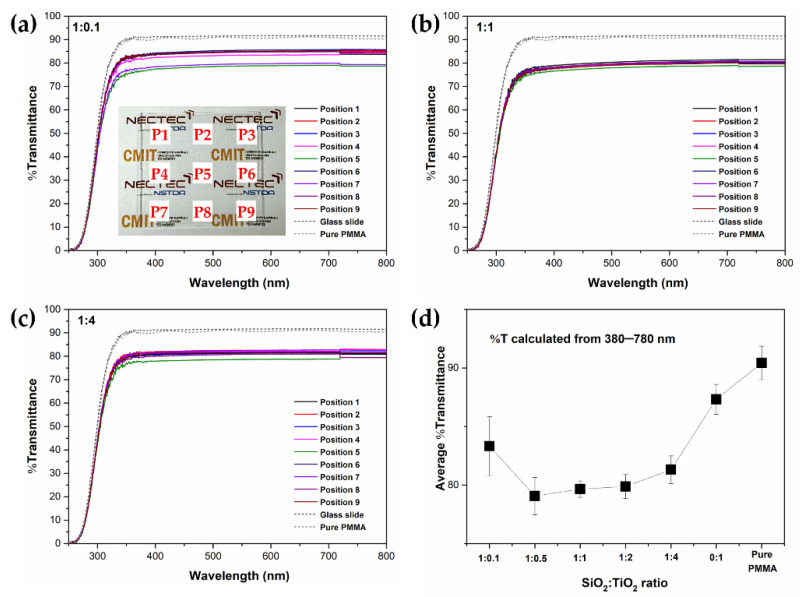
Transmittance spectra of SiO_2_/TiO_2_/PMMA composite films with different positions on film surface at various SiO_2_:TiO_2_ ratios (**a**) 1:0.1, (**b**) 1:1, (**c**) 1:4, and (**d**) %T average of all samples.

The transmission measurement of SiO_2_/TiO_2_/PMMA nanocomposite films was conducted on different positions on the film surface and the results are presented in Figure 6. The results show the flat spectra in the wavelength range of 380–780 nm without interference features and with a lower average transmittance than that of a bare glass substrate (90%), indicating the high transparency of the deposited films [22]. The decrease in transmission in the visible region of the specimen coated with SiO_2_/TiO_2_/PMMA composite film was regarded as related to the light-scattering phenomenon of the SiO_2_ and TiO_2_ particles embedded in the film. As seen in Figure 6a–c, the difference in their transmittance was additionally noticed at different measured positions on the sample’s surface. This result suggests a slight difference in film thickness on the whole covering surface and particle distribution over the whole surface due to the incorporation of a composite cluster and polymer matrix. In addition, all determined transmissions were reached as the wavelength was shorter than 380 nm due to typical absorption features of the bare glass substrate [22]. From the transmission spectra, the average transmission (%T_avg_) in the visible region was calculated using the equation expressed in Equation (2)

(2) $${\%T}_{\mathrm{avg}}=\frac{\int \phi_{\mathrm{lum}}\left(\lambda \right)T\left(\lambda \right)d\left(\lambda \right)}{\int \phi_{\mathrm{lum}}\left(\lambda \right)d\left(\lambda \right)},$$

where T(λ) is the transmittance at a specific wavelength (λ) and $\phi_{\mathrm{lum}}$ is the standard luminous efficiency function [23]. The calculated %T_avg_ at all areas was statistically determined, as shown in Figure 6d. The %T_avg_ tended to decrease with increasing TiO_2_ loading content up to the ratio of 1:1, and increased thereafter. This behavior could be attributed to the difference in particle distribution, as observed from the optical microscope.

**Figure 7:**
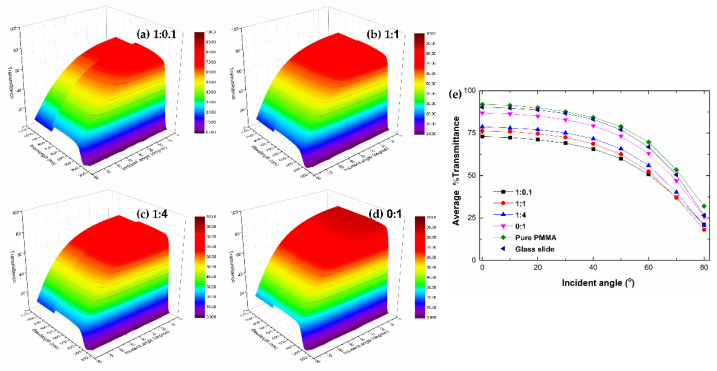
Omnidirectional transmittance of SiO_2_/TiO_2_/PMMA nanocomposite films with different SiO_2_:TiO_2_ ratios of (**a**) 1:0.1, (**b**) 1:1, (**c**) 1:4, (**d**) 0:1 and (**e**) the average transmission of SiO_2_/TiO_2_/PMMA nanocomposite films with different incident angles.

The omnidirectional transmittance of the prepared sample was investigated, as shown in Figure 7. The transmittance obviously decreased with the increase in the incident angle, following Snell’s law of refraction and Fresnel’s equations for reflection and transmission [24]. The transmission value of SiO_2_/TiO_2_/PMMA nanocomposite films as the incident angle ranged from 0° to 50° showed an insignificant change with an approximated %T = 80, indicating high transparency in the visible region. At greater incident angles beyond 60° and 80°, %transmission was less than 50% and 20%, respectively. Moreover, the extended width of the transmittance profile was improved by the influence of SiO_2_/TiO_2_ nanocomposite with the precursor ratio of 1:1. This result could be explained through the observation of the large particle distribution from the figures, as depicted in the optical microscope part. Meanwhile, the alternation of the refractive index on the top surface could be associated with the presence of SiO_2_/TiO_2_ particles, resulting in a significant reduction in transmission at a wide angle [24]. In addition, the step-like change in the spectra observed at 720 nm was affected by the change of filter within the spectrophotometer system during the measurement.

### 3.3. Self-Cleaning Surface Applications

**Figure 8:**
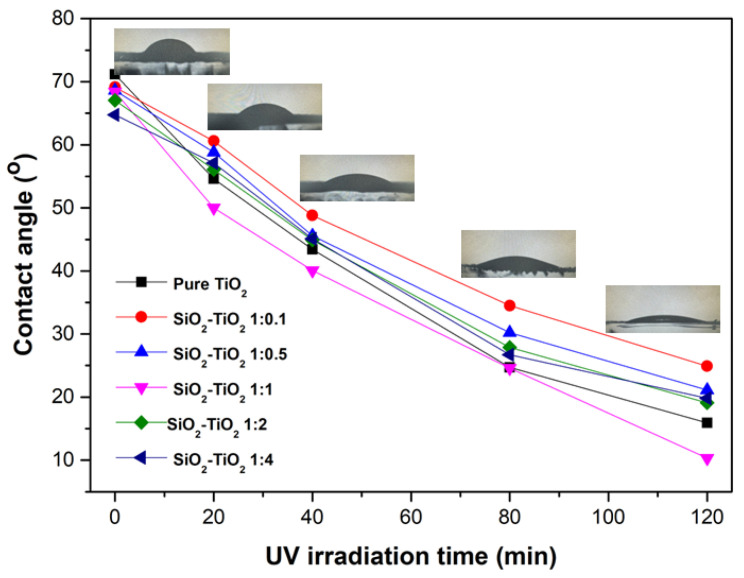
Water contact-angle measurement of SiO_2_/TiO_2_/PMMA nanocomposite films with different SiO_2_:TiO_2_ nanocomposite ratios.

For self-cleaning applications, the contact angle measurement interpreted by water droplets on SiO_2_/TiO_2_/PMMA nanocomposite films under UV irradiation for 120 min is illustrated in Figure 8. Before UV activation, the contact angle of a water droplet on each film was approximately 64–71°. After UV irradiation, the water flatting on all composite films was noticed, indicating the hydrophilic properties of the surface. The contact angle values of SiO_2_/TiO_2_/PMMA nanocomposite film with the increase in TiO_2_ loading in the SiO_2_ matrix were approximately 25.9°, 23.8°, 12.1°, 18.5°, and 21.0°, respectively. The contact angle value of TiO_2_/PMMA nanocomposite film was approximately 14.3°. An improvement in hydrophilicity was achieved when the film was incorporated with a SiO_2_/TiO_2_ composite with the specific ratio of 1:1, showing the lowest contact angle due to the good particle dispersion and homogeneity of the film structure. The presence of Si–O–Ti linkages originating from the SiO_2_/TiO_2_ nanocomposite relating to the increase in hydroxyl groups on the film surface could considerably improve hydrophilicity performance on the composite surface [25]. Moreover, the generation of electron–hole pairs from the TiO_2_ phase in the composite was a crucial mechanism, playing a key role in the hydrophilic mechanism under UV irradiation. The related mechanism is described. Electrons on the TiO_2_ surface were trapped by Ti(IV) cations Ti^4+^ to produce the Ti(III); Ti^3+^ state. Oxygen atoms were ejected and interacted with holes to create oxygen vacancies. Water molecules of the droplet on the film surface consequently occupied the oxygen vacancies to produce the adsorbed hydroxyl groups via H-bridging bonds with the water molecule. The stable formation of the Ti^3+^-OH functional group could possibly balance the TiO_2_ chemical structure, leading to a significant enhancement of hydrophilicity [26]. Under other conditions (SiO_2_:TiO_2_ at 1:0.1, 1:0.5, 1:2, and 1:4), high particle agglomeration of the composite and a large particle size are considered to be crucial parameters affecting the reduction in electron–hole pairs, hydroxyl groups, and the relation of Si–O–Ti linkages in the SiO_2_/TiO_2_ nanocomposite. Thus, the enhancement of hydrophilicity on the SiO_2_/TiO_2_/PMMA film surface is highly correlated with the number of hydroxyl groups and oxygen vacancies provided by the proper ratio of TiO_2_ being added to the SiO_2_ composite.

**Figure 9:**
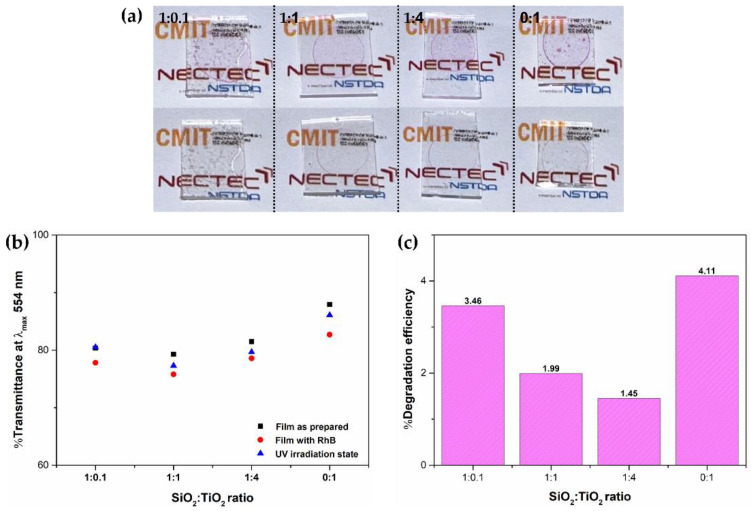
Photocatalytic performance with RhB dye under UV irradiation by SiO_2_/TiO_2_/PMMA composite films with different SiO_2_:TiO_2_ ratios, (**a**) the photographs of the composite films before and after UV irradiation, (**b**) %T values of RhB stain on the films at λ_max_ 554 nm, and (**c**) percentage degradation of RhB dye with SiO_2_/TiO_2_/PMMA composite films.

The photocatalytic activities of SiO_2_/TiO_2_/PMMA nanocomposite films with different SiO_2_:TiO_2_ ratios were evaluated according to rhodamine B (RhB) decomposition on the film surface, as shown in Figure 9. The photographs of SiO_2_/TiO_2_/PMMA composite films with RhB stain on the film surface taken before/after UV irradiation are shown in Figure 9a. Before UV exposure, the pink dye stain of the RhB droplet appeared on the film surface. After UV irradiation for 120 min, the dye stain was obviously removed by a PMMA/SiO_2_/TiO_2_ nanocomposite with a high TiO_2_ content in the composite up to the ratio of 1:1. To confirm dye degradation on the film surface, the evaluation of RhB transmission at λ_max_ 554 nm is illustrated in Figure 9b [27]. The photocatalytic reaction by means of RhB dye degradation on SiO_2_/TiO_2_/PMMA nanocomposite films with different SiO_2_:TiO_2_ ratios were studied in three situations: as-prepared film (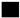), RhB stain on the composite film (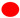), and after UV irradiation on RhB stain on the composite film (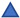). The transmission of as-prepared nanocomposite films was measured and used as a reference. For RhB droplets on the films, as shown in red dots, the decrease in %T value on the films is clearly noted due to the light absorbance of RhB dye. After UV irradiation, the %T values of all samples considerably increased due to the decrease in the dye absorbance or the diminished dye concentration, reflecting the effective RhB dye degradation by photocatalytic reaction on each film surface [28]. The highest photocatalytic degradation by the photocatalyst film was performed using pure TiO_2_ film due to its dominant photocatalytic property of anatase TiO_2_. Under UV irradiation, the electron–hole pairs created on the TiO_2_ photocatalyst surface strongly reacted with hydroxide groups and O_2_ molecules in the environment to produce the superoxide and hydroxyl radicals [29]. After that, the organic molecules in the RhB chemical structure were decomposed by these strong radicals to form in the conjugated chromophore, as presented by the clear RhB stain and higher %T value [30]. In the case of SiO_2_/TiO_2_/PMMA nanocomposite films, good photocatalytic activity was found in the sample with a SiO_2_:TiO_2_ ratio of 1:0.1. Although this specimen is mainly composed of SiO_2_ nanoparticles as a matrix, the photocatalytic reaction could be executed by the increase in active sites on the surface of SiO_2_ nanoparticles, providing a greater active surface area under UV irradiation. Moreover, the separation of photo-generated electron–hole pairs would be improved by the presence of oxygen defects on the SiO_2_ nanoparticle surface attributed to the decrease in the electron–hole pair recombination rate and enhancing the photocatalytic activity of the SiO_2_-based photocatalyst [31]. Meanwhile, the composite film with a 1:1 SiO_2_:TiO_2_ ratio had a higher photocatalytic reaction due to the homogenous particle dispersion on the film compared with the film with a SiO_2_:TiO_2_ ratio of 1:4. This reaction can be achieved due to the existence of a TiO_2_ photocatalyst in the SiO_2_/TiO_2_ nanocomposite. The photocatalytic reaction by the composite film with a SiO_2_:TiO_2_ ratio of 1:1 may be enhanced by the presence of a mixed TiOSi phase (Si–O–Ti linkages) at the TiO_2_/SiO_2_ interface, guiding the decrease in the tight agglomeration of the anatase phase in TiO_2_ material and the suppression of photoactive radicals by electron–hole pairs and effective surface with the silica phase in the composite. However, after UV irradiation, %T values of the composite films with SiO_2_:TiO_2_ ratios of 1:1, 1:4, and 0:1 were slightly different to the initial value of as-prepared films, which could be due to the coverage of well-dispersed particles on the film surface, as revealed by optical image analysis. A number of irradiation photons can be absorbed by the particles on the film surface, reflecting the decrease in %T value. Furthermore, the percentage of dye degradation in the film with RhB before and after UV irradiation was calculated to confirm the photocatalytic performance of the films, as shown in Figure 9c. The superior dye degradation efficiency of bare TiO_2_ film was determined to be approximately 4.11%, while the percentage of the SiO_2_/TiO_2_ composite films with SiO_2_:TiO_2_ ratios of 1:0.1, 1:1, and 1:4 was approximately 3.46, 1.99, and 1.45, respectively. According to the spherical shape of SiO_2_ nanoparticles in the composite film, a large number of active sites could be generated and enhance UV light absorption with their high surface-to-volume ratio, promoting the photocatalytic abilities under this condition. For the composite film with SiO_2_:TiO_2_ ratios at 1:1 and 1:4, the photocatalytic activity could be enhanced by the influence of the hydrophilicity of SiO_2_ and TiO_2_ according to the increase in the hydroxyl group generated on their surfaces.

## 4. Conclusions

SiO_2_/TiO_2_/PMMA nanocomposite films were fabricated using the spin-coating technique with the incorporation of an ultrafine SiO_2_/TiO_2_ composite with different TiO_2_ ratios in the PMMA polymer matrix. The thickness of the SiO_2_/TiO_2_/PMMA nanocomposite films was in the range of 486–666 nm, depending on the particle dispersion interpreted by transmission spectra. For self-cleaning, the composite film with a SiO_2_:TiO_2_ ratio of 1:1 showed a superior hydrophilic performance, showing the lowest contact angle of 12.1° after UV irradiation for 120 min, while the TiO_2_/PMMA nanocomposite film exhibited good photocatalytic activity by means of RhB dye degradation due to the preferential anatase phase of as-synthesized TiO_2_. Significant enhancements in the catalytic activity of the composite films with a 1:1 SiO_2_:TiO_2_ ratio are attributed to the good particle dispersion on the film surface with a greater active surface area and increasing active site radicals created by the TiO_2_ phase in the composite. It can be predicated that particle distribution, SiO_2_:TiO_2_ ratio, Si-O-Ti linkages induced at the interfaces, and the TiO_2_ phase in the composite are the crucial factors that play a key role in both the self-cleaning and photocatalytic properties of the films.

## Data Availability

The data presented in this study are available on request from the corresponding author.
